# Genetic diversity of rotavirus genome segment 6 (encoding VP6) in Pretoria, South Africa

**DOI:** 10.1186/2193-1801-3-179

**Published:** 2014-04-05

**Authors:** Martin M Nyaga, Mathew D Esona, Khuzwayo C Jere, Ina Peenze, Mapaseka L Seheri, M Jeffrey Mphahlele

**Affiliations:** Medical Research Council/Diarrhoeal Pathogens Research Unit, Department of Virology, Medunsa Campus, University of Limpopo/NHLS Dr George Mukhari Tertiary Laboratory, Pretoria, South Africa; Gastroenteritis and Respiratory Viruses Laboratory Branch, Division of Viral Diseases, NCIRD, CDC, Atlanta, Georgia USA; Department of Clinical Infection, Microbiology and Immunology, Institute of Infection and Global Health, University of Liverpool, Liverpool, UK

**Keywords:** Rotaviruses, Viral protein 6, Genetic diversity, South Africa, Pretoria

## Abstract

**Background:**

Rotavirus viral protein 6 (VP6), encoded by genome segment (GS) 6, is the primary target for rotavirus diagnosis by serological and some molecular techniques. Selected full length nucleotide sequences of GS 6 of rotavirus strains from South Africa were sequenced and analysed to determine genetic diversity and variations within the circulating rotaviruses.

**Findings:**

The VP6 amplicons were sequenced using the Sanger ABI 3130xl. Phylogenetic and pairwise analysis revealed that the VP6 genes of the study strains belonged to two different VP6 [I] genotypes. Five sequences were assigned genotype I1 and seven as genotype I2. Comparison of the group specific antigenic regions of the South African strains to the reference strains, shows that the South African VP6 sequences belonging to the VP6 genotype I2 were highly conserved, with only two amino acids changes at positions 239 (T›N) and 261(I›V). On the other hand, South African VP6 sequences belonging to I1 genotypes revealed several amino acid variations mostly within the antigenic region III.

**Conclusions:**

Rotavirus strains with I1 and I2 genotype are predominantly circulating within the South African communities of which the later seems to be more conserved within the antigenic regions. The observed genetic variations observed within GS 6 of rotaviruses analysed in the current study are unlikely to impact negatively on the performance of the current VP6-based detection methods. Nevertheless, investigators should continually consider this diversity and adapt the primer design for the detection and characterization of the VP6 gene accordingly.

**Electronic supplementary material:**

The online version of this article (doi:10.1186/2193-1801-3-179) contains supplementary material, which is available to authorized users.

## Findings

### Introduction

Rotaviruses form a genus rotavirus within the *Reoviridae* family (Estes and Kapikian 2007). Group A rotaviruses (RVA) are the major cause of severe dehydrating diarrhoea. Every year RVA infects approximately 114 million children that leads to 453,000 childhood deaths of which most occur in Africa and Asia (Tate et al. 2012). In South Africa, approximately 17,644 to 25,630 children are hospitalised of which 2,882 die annually due to rotavirus diarrhoea (WHO, 2012). An estimated 224 to 318 children die due to rotavirus disease in Pretoria and neighboring Brits areas of South Africa (Mapaseka et al. 2010).

RVA are non-enveloped tripled-layered enteric viruses that contain 11 double-stranded RNA GSs. The genome is encased within the VP2 (encoded by GS 2), inner VP6 (encoded by GS 6) capsid shell, and the outer glycosylated VP7 (encoded by GS 9) capsid that contains VP4 (encoded by GS 4). VP6 constitutes more than half of the mass of the rotavirus particle (Estes and Kapikian 2007) and contains rotavirus group antigens that are used to sub-classify RVA into subgroups (SG) I, II, I + II, and “non-I, non-II”) based on their reactivity to monoclonal antibodies (Greenberg et al. 1983).

Molecular methods have an edge in RVA diagnosis and classification over serological methods due to problems associated with the availability of monoclonal antibodies raised against specific rotavirus antigens. Of late, 16 genotypes (I1-I16) have been determined using a nucleotide sequence-based whole genome classification system (Matthijnssens et al. 2011). There is a correlation between the serological and genotype classifications where SG 1 and II corresponds to genotype 2 and 1, respectively (Iturriza-Gómara et al. 2002; Kerin et al. 2007; Matthijnssens et al. 2011).

GS 6 and its encoded VP6 play a major role in RVA detection. VP6 is the primary target antigen for RVA routine diagnostic serological techniques such as enzyme-linked immunosorbent assay (ELISA), immunofluorescence, and immunochromatography (Greenberg et al. 1983; Estes and Kapikian 2007). On the other hand, broadly reactive molecular diagnostic assays such as Reverse Transcriptase-Polymerase Chain Reaction (RT)-PCR protocols targets the ends or internal conserved regions of GS 6 nucleotide sequences by employing hybridization methods which uses sequence specific primers and/or probes (Iturriza-Gómara et al. 2002; Lin et al. 2008). The first molecular assays that were developed in the early 1990s utilised GS 6 nucleotide sequences of rotavirus strains circulating from early 1970s to late 1980s (Grinde et al. 1995). More recently, RT-PCR assays based on GS6 have also been developed using old reference and a few new human rotavirus GS 6 sequences. This resulted in a more reliable scheme for conveying various VP6 genogroups of human rotaviruses (Lin et al. 2008; Matthijnssens et al. 2012).

The availability of many complete GS 6 sequences in the GenBank database will ease the prior challenges of improving, developing and validating rotavirus characterisation methods that utilises VP6 encoding GS (Lin et al. 2008). It is important to continuously evaluate the nucleotide sequences for GS 6 of strains circulating particularly in African countries owing to the wide rotavirus G and P genotype variations that have been consistently reported (Mwenda et al. 2010; Kiulia et al. 2014; Seheri et al. 2014; Tsolenyanu et al. 2014). Such studies would inform in advance, potential evolutionary variations that would eventually affect efficiency of VP6 based rotavirus characterisation methods. In this report, the genetic variation of the GS 6 of rotavirus strains collected recently in Pretoria, South Africa, were characterised and compared against those of strains reported from other parts of the world.

## Materials and methods

### Ethical consideration and sample selection

The project was approved by the Medunsa Research Ethics Committee (MREC/P/168/2008; MREC/P/108/2013; PG). Eleven human stool specimens were selected randomly from previously screened ELISA rotavirus-positive samples from children presenting with diarrhoea at Lancet private pathology laboratories, Pretoria in 2008. These samples included two commonly characterised G1P[8], six uncommon G and P genotype combinations (G1P[4] and G2P[6]), and three G9P[8]/G9P[6] strains that have emerged in the last two decades. One rotavirus ELISA rotavirus-positive porcine sample obtained from the archive of the Medical Research Council-Diarrhoeal Pathogens Research Unit (MRC-DPRU) laboratory that could not be assigned G and P genotypes was also analysed. Nucleotide sequences for human and animal rotavirus strains containing G2, G8, G9 and G12 were selected from our previous studies (Jere et al. 2011,2012,2014; Nyaga et al. 2013) to cover frequently characterised strains around this region.

### VP7, VP4 and VP6 RT-PCR and genotyping of rotavirus strains

Rotavirus dsRNA was extracted from 10% fecal supernatant using the QIAamp viral extraction kit by following the manufacturer’s instructions (Qiagen, Valencia, CA). Synthesis of cDNA and genotyping of the GS 4 and 9 were performed using RT-PCR as described previously (Góuvea et al. 1990; Gentsch et al. 1992; Iturriza-Gómara et al. 2001; Iturriza-Gómara et al. 2004). In-house designed primer set VP6F (nt 1–20, 5’GGCTTTAAAACGAAGTCTTC3’) and VP6R (nt 1336–1356, 5’TGTAGTGAGAGGATGTGACC 3’) was used to amplify the entire GS 6 (1356 bp). In brief, RNA was denatured at 95°C for 5 min followed by reverse transcription at 50°C for 30 min. Thirty PCR cycles of 95°C for 30 sec, 50°C for 30 sec and 72°C for 45 sec, followed by an elongation step at 72°C for 7 min was performed. The PCR products were purified with the QIAquick PCR Purification Kit (Qiagen, Valencia, CA) by following the manufacturer’s instructions. The PCR amplicon were run on a 1% Tris-acetate- ethylenediaminetetraacetic acid- agarose gel stained with 1% ethidium bromide. The GS 6 cDNA amplicons were sequenced using the Sanger ABI 3130xl at Inqaba Biotechnical Industries (Pty) Ltd, Pretoria, South Africa. RotaC [http://rotac.regatools.be/] (Maes et al. 2009) was used to assign genotypes to GS 6.

### Phylogenetic method

Sequences were aligned using the MUSCLE program within MEGA version 5 (Tamura et al. 2011). Once aligned, the JModelTest 2 program (Posada 2008) was used to identify the optimal evolutionary model that best fitted the sequence datasets. Using corrected Akaike Information Criterion (AICc) the following model; TN93 + G + I was found to best fit the sequence data for the VP6 gene. Using these models, maximum likelihood trees were constructed using MEGA version 5 with 500 bootstrap replicates to estimate branch support. Nucleotide and amino acid distance matrixes were prepared using the *p*-distance algorithm in MEGA version 5 (Tamura et al. 2011).

## Results

The complete sequences of the GS 6 of each selected strain contained 1356 nt and a single ORF from nt 24 to 1214 resulting in deduced protein of 397 amino acid. These were submitted to the GenBank under accession numbers KC618421-KC618432.

**Figure 1:**
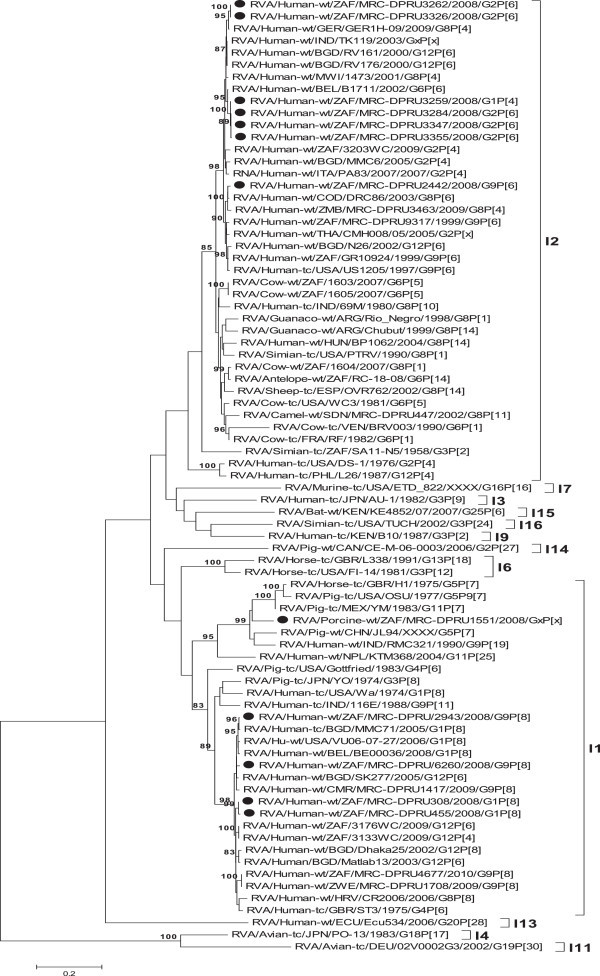
**Maximum likelihood phylogenetic trees built in MEGA version 5 with bootstrap statistics as support, show the genetic relationships of nucleotide sequences of VP6 of human and porcine strains from South Africa with known human and animal rotavirus VP6 sequences from the GenBank database.** The tree was drawn to scale. Only bootstrap values of 80% and greater are shown. Bars represent 0.2 substitutions per nucleotide position. South African study strains are indicated by filled circle.

GS 6 of the study strains were assigned either I1 or I2 genotypes as indicated in Additional file 1: Table S1. All the South Africa strains with genotype combination of G1P[8] or G9P[8] were assigned VP6 genotype I1 and they shared a nucleotide (amino acid) identity in the range of 82.8-99.6% (91.7-99%), while those with G2P[6] or G1P[4] or G9P[6], were closely related to strains belonging to genotype I2 and shared a nucleotide (amino acid) identity in the range of 79.2-100% (91.2-100%). The South African VP6 genotype I1 strains analysed in this study shared a very high nucleotide (amino acid) identity in the range of 81.1-99.7% (91.4-99.7%) with both reference human (such as RVA/Human-tc/USA/Wa/1974/G1P[8]) and animal (such as RVA/Pig-tc/USA/OSU/1977/G5P9[7]) genotype I1 VP6 sequences obtained from the GenBank database. On the other hand, South African strains assigned as VP6 genotype I2 also shared a nucleotide (amino acid) identity in the range of 86.6-99% (96.2-99.7%) to both reference human (such as RVA/Human-tc/USA/DS-1/1976/G2P[4]) and animal (such as RVA/Sheep-tc/ESP/OVR762/2002/G8P[14]) strains in the same genotype (Additional file 1: Table S1). Phylogenetic analysis divides the South African strains into two separate clusters (Figure 1). Consistent with distance analyses, South African strains in genotype I1 formed a phylogenetic cluster with human rotavirus SGII (Wa-like or genotype I1) strains, while the remaining in genotype I2 clustered with DS-1-like or I2 genotype (SGI) GS 6.

Comparison of the amino acid sequences of VP6 proteins of South African strains determined in this study and those from our previous studies, couple with some human and animal rotavirus reference strains reveals several regions that are completely or highly conserved among the South African GS 6 sequences compared to their respective genotypes (Additional file 2). However, South African strains belonging to the I1 or I2 genotypes were closely related to Wa-like or DS-1-like strains, respectively (Additional file 1: Table S1 and Figure 1). Comparison of the group specific antigenic regions of the South African strains to the reference strain RF, shows that the South African VP6 sequences belonging to the VP6 genotype I2 were highly conserved, with only two amino acids changes at positions 239 (T›N) and 261(I›V). On the other hand, South African VP6 sequences belonging to I1 genotypes revealed eight amino acid changes at positions 39(I›V), 45(E›D), 60(N›T), 239 (T›N), 248(Y›F), 252(V›I), 261(I›V) and 396 (V›I). Also, the Subgroup I (SGI) and Subgroup II (SGII) residues were highly conserved among South African strains belonging to I2 genotype, while amino acid changes were observed in SGI residues at positions 172 (A›M), 305 (A›N) and 310 (N›Q) of South African strain in I1 genotype.

## Discussion and conclusions

The GS 6 in this study showed significant amounts of genetic variation among strains within the two genotypes. The fact that strains within the I2 genotype were more conserved with less amino acid changes than the I1 strains was supported by the differences in the nucleotide and deduced amino acid identities. For instance, the nucleotide changes for genotype I1 diverged by nt (aa) 3.1 (0.5) and 18.0 (8.6) between themselves and when compared to reference sequences from the GenBank, respectively. Those of I2 diverged by a wider margin, nt (aa) 17.2 (8.3) and 18.0 (8.6) between themselves and when compared to reference sequences from the GenBank, respectively. This could mean that in Pretoria, South Africa, genotype I1 is more prone to genetic changes than genotype I2 and could potentially result in detection inconsistencies in future. The effect of the observed genetic variations on the host disease presentation warrants further understanding.

In these analyses, the GS 6 of previously analysed local circulating strains were also included (Jere et al. 2011,2012,2014; Nyaga et al. 2013). Like any other rotavirus GS, the VP6 encoding GS 6 from these strains could be swiftly evolving at different rates due to antigenic drifts caused by point mutations and was clearly shown by the clustering of strain RVA/Human-wt/ZAF/MRC-DPRU3259/2008/G1P[4] with I2 strains. The G1s have previously been reported as only I1 strains (Ghosh and Kobayashi 2011). The nucleotide and amino acid changes reported here were consisted with other studies (Johne et al. 2011; Matthijnssens et al. 2012). Changes occurring at the nucleotide level of VP6-encoding gene over time could cause amino acid sequence variations resulting in changes in VP6 epitopes. This could potentially affect the efficiency of the rotavirus detection methods (Kerin et al. 2007).

The current VP6 detection techniques should work efficiently as the changes observed in the antigenic regions of these South African strains seems not to vary significantly. The limitation of this study is the small sample size albeit including various distinct strains, hence a conclusively determination on how rare or common these changes are occurring in Pretoria, South Africa, cannot be made. Although the sample size was small, the findings were consistent with those reported elsewhere (Kerin et al. 2007; Johne et al. 2011; Matthijnssens et al. 2012). The results suggest that rotavirus investigators should continually consider this diversity and update the primer required for the detection and characterization of the VP6 gene accordingly.

## Electronic supplementary material

Additional file 1: Table S1: Nucleotide (NT) and amino acid (AA) percentage identities of South Africa VP6 sequences compared to VP6 sequences of human and animal obtained from the GenBank. (PDF 73 KB)

Additional file 2: **Comparison of deduced amino acid sequences of VP6 proteins of South African strains to strain RF.** Gray areas indicates boundaries of group specific antigenic regions (antigenic site I (32-64), antigenic site II (155-167), antigenic site III (208-274) and antigenic site IV (380-397). Residues 228-240 (Gray box area) indicates hyper variable region, residues 172, 296-300, 305 and 310 (plain box) are Subgroup I (SGI) and residues at position 306 is a Subgroup II (SGII). (DOC 145 KB)

## Abbreviations

VP: Viral protein
GS: Genome segment
RVA: Group A rotaviruses
WHO: World Health Organization
RNA: Ribonucleic acid
SG: Subgroup
ELISA: Enzyme linked immunosorbent assay
RT-PCR: Reverse transcriptase polymerase chain reaction
MREC: Medunsa Research Ethics Committee
MRC: Medical research council
DPRU: Diarrhoeal Pathogens Research Unit
cDNA: complementary Deoxyribonucleic acid
kb: Kilobase
AICs: Akaike Information Criterion
nt: Nucleotide
aa: Amino acid.
