# Supplementary material for: Genetic diversity of rotavirus genome segment 6 (encoding VP6) in Pretoria, South Africa
Source: Springerplus. 2014 Apr 5;3:179. doi: 10.1186/2193-1801-3-179 (PMC4000354; doi:10.1186/2193-1801-3-179)
Supplement: Supplementary file 1 — Additional file 1: Table S1: Nucleotide (NT) and amino acid (AA) percentage identities of South Africa VP6 sequences compared to VP6 sequences of human and animal obtained from the GenBank. (PDF 73 KB) [file 40064_2013_888_MOESM1_ESM.pdf]

Table 1: Nucleotide (NT) and amino acid (AA) percentage identities of South Africa VP6 sequences (in box) compared to VP6 sequences of human and animal obtained from the Genbank.

**Strain**	**VP6**	DPRU308		DPRU455		DPRU2943		DPRU6260		DPRU1551		DPRU2442		DPRU3259		DPRU3284		DPRU3347		DPRU3355		DPRU3262		DPRU3326	
	**Genotype**	NT	**AA**	NT	**AA**	NT	**AA**	NT	**AA**	NT	**AA**	NT	**AA**	NT	**AA**	NT	**AA**	NT	**AA**	NT	**AA**	NT	**AA**	NT	**AA**
**RVA/Human-wt/ZAF/MRC-DPRU308/2008/G1P[8]**	**I1**																								
**RVA/Human-wt/ZAF/MRC-DPRU455/2008/G1P[8]**	**I1**	99.6	**98.7**																						
**RVA/Human-wt/ZAF/MRC-DPRU/2943/2008/G9P[8]**	**I1**	97.4	**99.0**	97.0	**97.7**																				
**RVA/Human-wt/ZAF/MRC-DPRU/6260/2008/G9P[8]**	**I1**	97.4	**99.0**	97.0	**97.7**	98.4	**98.7**																		
**RVA/Porcine-wt/ZAF/MRC-DPRU1551/2008/GxP[x]**	**I1**	83.2	**92.9**	82.8	**91.7**	83.3	**93.2**	83.3	**92.9**																
**RVA/Human-wt/ZAF/MRC-DPRU2442/2008/G9P[6]**	**I2**	79.6	**92.4**	79.2	**91.2**	79.8	**92.7**	80.0	**92.7**	79.9	**91.4**														
**RVA/Human-wt/ZAF/MRC-DPRU3259/2008/G1P[4]**	**I2**	79.9	**92.4**	79.5	**91.2**	79.8	**92.7**	80.4	**92.7**	79.9	**91.4**	97.2	**100**												
**RVA/Human-wt/ZAF/MRC-DPRU3284/2008/G2P[6]**	**I2**	79.9	**92.7**	79.5	**91.4**	79.8	**92.9**	80.4	**92.9**	80.1	**91.7**	97.0	**99.7**	99.7	**99.7**										
**RVA/Human-wt/ZAF/MRC-DPRU3347/2008/G2P[6]**	**I2**	79.9	**92.7**	79.5	**91.4**	79.8	**92.9**	80.4	**92.9**	80.1	**91.7**	97.0	**99.7**	99.7	**99.7**	100	**100**								
**RVA/Human-wt/ZAF/MRC-DPRU3355/2008/G2P[6]**	**I2**	79.8	**92.4**	79.4	**91.2**	79.8	**92.7**	80.4	**92.7**	80.0	**91.4**	96.9	**99.5**	99.7	**99.5**	99.9	**99.7**	99.9	**99.7**						
**RVA/Human-wt/ZAF/MRC-DPRU3262/2008/G2P[6]**	**I2**	80.7	**92.4**	80.3	**91.2**	80.6	**92.7**	81.1	**92.7**	80.1	**91.4**	97.1	**100**	98.1	**100**	98.0	**99.7**	98.0	**99.7**	97.9	**99.5**				
**RVA/Human-wt/ZAF/MRC-DPRU3326/2008/G2P[6]**	**I2**	80.7	**92.4**	80.3	**91.2**	80.6	**92.7**	81.1	**92.7**	80.1	**91.4**	97.1	**100**	98.1	**100**	98.0	**99.7**	98.0	**99.7**	97.9	**99.5**	100	**100**		
RVA/Human-wt/ZAF/3176WC/2009/G12P[6]	I1	98.2	**99.2**	97.8	**98.0**	97.2	**99.0**	97.1	**99.2**	83.4	**93.2**	79.8	**92.7**	80.1	**92.7**	80.1	**92.9**	80.1	**92.9**	80.0	**92.7**	80.9	**92.7**	80.9	**92.7**
RVA/Human-wt/ZAF/MRC-DPRU4677/2010/G9P[8]	I1	97.6	**99.5**	97.2	**98.2**	96.6	**99.2**	96.7	**99.5**	83.4	**93.5**	79.6	**92.9**	79.8	**92.9**	79.9	**93.2**	79.9	**93.2**	79.8	**92.9**	80.4	**92.9**	80.4	**92.9**
RVA/Human-wt/ZWE/MRC-DPRU1708/2009/G9P[8]	I1	97.6	**99.5**	97.2	**98.2**	96.6	**99.2**	96.7	**99.5**	83.4	**93.5**	79.6	**92.9**	79.8	**92.9**	79.9	**93.2**	79.9	**93.2**	79.8	**92.9**	80.4	**92.9**	80.4	**92.9**
RVA/Human-wt/HRV/CR2006/2006/G8P[8]	I1	96.2	**98.0**	95.8	**97.0**	95.8	**97.7**	95.7	**98.0**	83.0	**91.9**	78.8	**91.4**	78.7	**91.4**	78.8	**91.7**	78.8	**91.7**	78.7	**91.4**	79.6	**91.4**	79.6	**91.4**
RVA/Pig-tc/MEX/YM/1983/G11P[7]	I1	82.5	**92.7**	82.0	**91.4**	82.8	**92.9**	83.0	**92.7**	90.3	**98.7**	80.4	**91.2**	80.0	**91.2**	80.0	**91.4**	80.0	**91.4**	80.1	**91.2**	80.4	**91.2**	80.4	**91.2**
RVA/Pig-wt/CHN/JL94/XXXX/G5P[7]	I1	82.0	**92.7**	81.6	**91.4**	82.1	**92.9**	82.0	**92.9**	90.0	**97.5**	79.6	**91.2**	79.3	**91.2**	79.5	**91.4**	79.5	**91.4**	79.6	**91.2**	79.6	**91.2**	79.6	**91.2**
RVA/Pig-tc/USA/Gottfried/1983/G4P[6]	I1	88.8	**97.5**	88.4	**96.2**	88.1	**97.7**	88.2	**97.5**	83.6	**93.5**	81.7	**93.5**	81.7	**93.5**	81.8	**93.7**	81.8	**93.7**	81.7	**93.5**	82.0	**93.5**	82.0	**93.5**
RVA/Pig-tc/JPN/YO/1974/G3P[8]	I1	89.7	**97.5**	89.3	**96.2**	89.7	**97.2**	89.8	**97.5**	84.0	**94.2**	80.3	**92.9**	80.9	**92.9**	81.0	**93.2**	81.0	**93.2**	80.9	**92.9**	80.7	**92.9**	80.7	**92.9**
RVA/Human-wt/ZAF/3133WC/2009/G12P[4]	I1	98.2	**99.2**	97.8	**98.0**	97.2	**99.0**	97.1	**99.2**	83.4	**93.2**	79.8	**92.7**	80.1	**92.7**	80.1	**92.9**	80.1	**92.9**	80.0	**92.7**	80.9	**92.7**	80.9	**92.7**
RVA/Human-wt/NPL/KTM368/2004/G11P[25]	I1	83.3	**92.9**	82.9	**91.7**	83.6	**93.2**	83.9	**92.9**	84.6	**93.7**	78.8	**89.7**	79.2	**89.7**	79.3	**89.9**	79.3	**89.9**	79.3	**89.7**	79.0	**89.7**	79.0	**89.7**
RVA/Human-tc/USA/Wa/1974/G1P[8]	I1	89.9	**97.2**	89.5	**96.0**	89.2	**97.0**	89.4	**97.2**	82.6	**92.9**	79.4	**92.2**	80.0	**92.2**	80.1	**92.4**	80.1	**92.4**	80.0	**92.2**	79.9	**92.2**	79.9	**92.2**
RVA/Human-tc/IND/116E/1988/G9P[11]	I1	90.5	**97.7**	90.1	**96.5**	89.8	**97.5**	90.2	**97.7**	82.9	**93.2**	80.8	**92.7**	80.9	**92.7**	81.2	**92.9**	81.2	**92.9**	81.1	**92.7**	81.2	**92.7**	81.2	**92.7**
RVA/Human-tc/GBR/ST3/1975/G4P[6]	I1	97.2	**99.2**	96.8	**98.0**	96.4	**99.0**	96.3	**99.2**	83.3	**93.2**	79.3	**92.7**	79.5	**92.7**	79.6	**92.9**	79.6	**92.9**	79.5	**92.7**	80.1	**92.7**	80.1	**92.7**
RVA/Human-wt/BGD/Dhaka25/2002/G12P[8]	I1	97.6	**99.5**	97.1	**98.2**	96.9	**99.2**	96.6	**99.5**	84.0	**93.5**	80.1	**92.9**	80.3	**92.9**	80.3	**93.2**	80.3	**93.2**	80.2	**92.9**	80.9	**92.9**	80.9	**92.9**
RVA/Horse-tc/GBR/H1/1975/G5P[7]	I1	81.6	**92.9**	81.2	**91.7**	82.0	**93.2**	82.1	**92.9**	89.0	**97.7**	80.1	**92.2**	79.8	**92.2**	79.8	**92.4**	79.8	**92.4**	79.9	**92.2**	80.1	**92.2**	80.1	**92.2**
RVA/Human/BGD/Matlab13/2003/G12P[6]	I1	97.9	**99.5**	97.5	**98.2**	97.1	**99.2**	96.8	**99.5**	83.8	**93.5**	79.8	**92.9**	80.0	**92.9**	80.0	**93.2**	80.0	**93.2**	79.9	**92.9**	80.8	**92.9**	80.8	**92.9**
RVA/Human-wt/IND/RMC321/1990/G9P[19]	I1	82.7	**92.7**	82.3	**91.4**	82.8	**92.9**	82.9	**92.7**	90.2	**98.2**	80.4	**90.9**	79.8	**90.9**	80.0	**91.2**	80.0	**91.2**	80.1	**90.9**	79.9	**90.9**	79.9	**90.9**
RVA/Pig-tc/USA/OSU/1977/G5P9[7]	I1	81.5	**93.2**	81.1	**91.9**	81.9	**93.5**	82.0	**93.2**	88.1	**98.7**	79.4	**91.9**	79.1	**91.9**	79.3	**92.2**	79.3	**92.2**	79.3	**91.9**	79.5	**91.9**	79.5	**91.9**
RVA/Human-tc/BGD/MMC71/2005/G1P[8]	I1	97.1	**99.0**	96.7	**97.7**	99.7	**99.7**	98.2	**99.0**	83.3	**93.5**	79.7	**92.9**	79.7	**92.9**	79.7	**93.2**	79.7	**93.2**	79.6	**92.9**	80.4	**92.9**	80.4	**92.9**
RVA/Hu-wt/USA/VU06-07-27/2006/G1P[8]	I1	97.4	**98.7**	97.0	**97.5**	99.2	**99.5**	98.2	**98.7**	83.6	**93.2**	79.7	**92.7**	79.8	**92.7**	79.8	**92.9**	79.8	**92.9**	79.8	**92.7**	80.6	**92.7**	80.6	**92.7**
RVA/Human-wt/BEL/BE00036/2008/G1P[8]	I1	97.1	**99.0**	96.7	**97.7**	99.2	**99.7**	98.2	**99.0**	83.8	**93.5**	79.9	**92.9**	80.1	**92.9**	80.1	**93.2**	80.1	**93.2**	80.0	**92.9**	80.9	**92.9**	80.9	**92.9**
RVA/Human-wt/BGD/SK277/2005/G12P[6]	I1	97.9	**99.5**	97.5	**98.2**	98.7	**99.2**	98.7	**99.5**	83.9	**93.5**	80.3	**92.9**	80.4	**92.9**	80.4	**93.2**	80.4	**93.2**	80.4	**92.9**	81.2	**92.9**	81.2	**92.9**
RVA/Human-wt/CMR/MRC-DPRU1417/2009/G9P[8]	I1	97.4	**99.0**	97.0	**97.7**	98.4	**98.7**	98.3	**99.0**	83.6	**93.2**	80.1	**92.7**	80.3	**92.7**	80.3	**92.9**	80.3	**92.9**	80.2	**92.7**	81.0	**92.7**	81.0	**92.7**
RVA/Cow-wt/ZAF/1604/2007/G8P[1]	I2	80.0	**91.9**	79.6	**90.7**	80.1	**92.2**	80.1	**92.2**	79.3	**90.9**	93.9	**99.5**	93.4	**99.5**	93.3	**99.2**	93.3	**99.2**	93.2	**99.0**	93.7	**99.5**	93.7	**99.5**
RVA/Simian-tc/ZAF/SA11-N5/1958/G3P[2]	I2	78.8	**92.2**	78.4	**90.9**	79.1	**92.4**	78.6	**92.4**	80.2	**91.4**	86.6	**97.7**	86.6	**97.7**	86.6	**97.5**	86.6	**97.5**	86.6	**97.2**	86.7	**97.7**	86.7	**97.7**
RVA/Camel-wt/SDN/MRC-DPRU447/2002/G8P[11]	I2	80.7	**91.7**	80.3	**90.4**	80.0	**91.9**	80.2	**91.9**	79.4	**90.2**	92.9	**98.7**	92.5	**98.7**	92.4	**98.5**	92.4	**98.5**	92.4	**98.2**	93.5	**98.7**	93.5	**98.7**
RVA/Cow-wt/ZAF/1603/2007/G6P[5]	I2	79.1	**91.7**	78.7	**90.4**	79.1	**91.9**	79.1	**91.9**	79.3	**91.4**	93.4	**98.7**	93.3	**98.7**	93.4	**99.0**	93.4	**99.0**	93.3	**98.7**	93.5	**98.7**	93.5	**98.7**
RVA/Cow-wt/ZAF/1605/2007/G6P[5]	I2	79.1	**91.7**	78.7	**90.4**	79.1	**91.9**	79.1	**91.9**	79.3	**91.4**	93.4	**98.7**	93.3	**98.7**	93.4	**99.0**	93.4	**99.0**	93.3	**98.7**	93.5	**98.7**	93.5	**98.7**
RVA/Antelope-wt/ZAF/RC-18-08/G6P[14]	I2	79.9	**91.9**	79.5	**90.7**	80.0	**92.2**	80.0	**92.2**	79.8	**90.9**	94.0	**99.5**	93.4	**99.5**	93.3	**99.2**	93.3	**99.2**	93.2	**99.0**	93.9	**99.5**	93.9	**99.5**
RVA/Human-wt/MWI/1473/2001/G8P[4]	I2	79.6	**91.9**	79.2	**90.7**	79.5	**92.2**	80.0	**92.2**	79.8	**90.9**	97.0	**99.5**	98.0	**99.5**	97.9	**99.2**	97.9	**99.2**	97.8	**99.0**	98.1	**99.5**	98.1	**99.5**
RVA/Guanaco-wt/ARG/Rio_Negro/1998/G8P[1]	I2	80.4	**91.7**	80.0	**90.4**	80.2	**91.9**	80.4	**91.9**	79.1	**90.7**	92.0	**99.2**	91.7	**99.2**	91.6	**99.0**	91.6	**99.0**	91.5	**98.7**	92.9	**99.2**	92.9	**99.2**
RVA/Human-wt/HUN/BP1062/2004/G8P[14]	I2	80.6	**92.4**	80.2	**91.2**	80.6	**92.7**	80.8	**92.7**	80.3	**91.4**	92.6	**99.5**	92.5	**99.5**	92.6	**99.2**	92.6	**99.2**	92.5	**99.0**	93.4	**99.5**	93.4	**99.5**
RVA/Guanaco-wt/ARG/Chubut/1999/G8P[14]	I2	79.2	**91.7**	78.8	**90.4**	79.0	**91.9**	79.3	**91.9**	79.5	**90.7**	92.4	**99.2**	92.3	**99.2**	92.4	**99.0**	92.4	**99.0**	92.3	**98.7**	92.6	**99.2**	92.6	**99.2**
RVA/Human-wt/ZAF/3203WC/2009/G2P[4]	I2	79.5	**91.9**	79.1	**90.7**	79.5	**92.2**	79.7	**92.2**	79.8	**90.9**	96.4	**99.5**	97.0	**99.5**	96.9	**99.2**	96.9	**99.2**	96.8	**99.0**	96.9	**99.5**	96.9	**99.5**
RVA/Human-wt/GER/GER1H-09/2009/G8P[4]	I2	80.1	**92.2**	79.7	**90.9**	79.9	**92.4**	80.4	**92.4**	79.8	**91.2**	96.6	**99.7**	97.6	**99.7**	97.6	**99.5**	97.6	**99.5**	97.5	**99.2**	99.2	**99.7**	99.2	**99.7**
RVA/Human-tc/IND/69M/1980/G8P[10]	I2	79.9	**91.4**	79.5	**90.2**	79.8	**91.7**	80.0	**91.7**	79.7	**90.4**	93.5	**99.0**	92.9	**99.0**	92.8	**98.7**	92.8	**98.7**	92.7	**98.5**	93.5	**99.0**	93.5	**99.0**
RVA/Human-wt/ZAF/MRC-DPRU9317/1999/G9P[6]	I2	79.8	**91.7**	79.3	**90.4**	79.6	**91.9**	80.1	**91.9**	79.8	**90.7**	97.6	**99.2**	97.1	**99.2**	97.0	**99.0**	97.0	**99.0**	96.9	**98.7**	96.8	**99.2**	96.8	**99.2**
RVA/Simian-tc/USA/PTRV/1990/G8P[1]	I2	79.5	**91.9**	79.1	**90.7**	79.8	**92.2**	79.9	**92.2**	79.9	**90.9**	92.8	**99.5**	91.9	**99.5**	92.0	**99.2**	92.0	**99.2**	91.9	**99.0**	92.3	**99.5**	92.3	**99.5**
RVA/Sheep-tc/ESP/OVR762/2002/G8P[14]	I2	79.8	**91.7**	79.3	**90.4**	79.8	**91.9**	79.5	**91.9**	79.4	**90.2**	93.1	**98.7**	92.5	**98.7**	92.6	**98.5**	92.6	**98.5**	92.5	**98.2**	93.0	**98.7**	93.0	**98.7**
RVA/Human-wt/ZAF/GR10924/1999/G9P[6]	I2	79.7	**92.2**	79.3	**90.9**	79.5	**92.2**	79.9	**91.9**	79.6	**90.7**	97.7	**99.2**	97.1	**99.2**	97.1	**99.0**	97.1	**99.0**	97.0	**98.7**	97.1	**99.2**	97.1	**99.2**
RVA/Human-wt/COD/DRC86/2003/G8P[6]	I2	79.1	**91.9**	78.7	**90.7**	79.3	**92.2**	79.5	**92.2**	79.6	**90.9**	99.0	**99.5**	96.7	**99.5**	96.5	**99.2**	96.5	**99.2**	96.4	**99.0**	96.8	**99.5**	96.8	**99.5**
RVA/Human-wt/BGD/RV161/2000/G12P[6]	I2	79.8	**92.2**	79.3	**90.9**	79.7	**92.4**	80.2	**92.4**	80.1	**91.2**	97.2	**99.7**	98.3	**99.7**	98.2	**99.5**	98.2	**99.5**	98.2	**99.2**	98.7	**99.7**	98.7	**99.7**
RVA/Human-wt/BGD/N26/2002/G12P[6]	I2	80.0	**91.9**	79.6	**90.7**	79.8	**91.9**	80.3	**91.7**	79.8	**90.4**	97.1	**99.0**	96.5	**99.0**	96.4	**98.7**	96.4	**98.7**	96.3	**98.5**	96.9	**99.0**	96.9	**99.0**
RVA/Human-wt/BGD/MMC6/2005/G2P[4]	I2	79.6	**91.7**	79.2	**90.4**	79.8	**91.9**	80.2	**91.9**	79.8	**90.7**	96.5	**99.2**	97.1	**99.2**	97.0	**99.0**	97.0	**99.0**	96.9	**98.7**	97.0	**99.2**	97.0	**99.2**
RVA/Human-tc/USA/DS-1/1976/G2P[4]	I2	81.3	**92.7**	80.9	**91.4**	81.4	**92.7**	81.0	**92.4**	80.3	**91.2**	87.4	**98.5**	87.4	**98.5**	87.4	**98.2**	87.4	**98.2**	87.3	**98.0**	87.6	**98.5**	87.6	**98.5**
RVA/Human-tc/PHL/L26/1987/G12P[4]	I2	81.4	**92.2**	80.9	**90.9**	81.0	**92.2**	81.0	**91.9**	80.5	**90.7**	87.1	**97.7**	87.2	**97.7**	87.1	**97.5**	87.1	**97.5**	87.0	**97.2**	87.4	**97.7**	87.4	**97.7**
RVA/Cow-tc/VEN/BRV003/1990/G6P[1]	I2	78.5	**90.9**	78.1	**89.7**	78.8	**91.2**	78.7	**91.2**	76.8	**89.2**	87.9	**96.5**	87.6	**96.5**	87.4	**96.2**	87.4	**96.2**	87.3	**96.0**	87.5	**96.5**	87.5	**96.5**
RVA/Cow-tc/USA/WC3/1981/G6P[5]	I2	80.5	**91.7**	80.1	**90.4**	80.3	**91.9**	80.4	**91.9**	79.9	**91.2**	93.9	**99.2**	93.5	**99.2**	93.5	**99.0**	93.5	**99.0**	93.4	**98.7**	93.7	**99.2**	93.7	**99.2**
RVA/Cow-tc/FRA/RF/1982/G6P[1]	I2	80.1	**91.2**	79.7	**89.9**	79.9	**91.4**	80.3	**91.4**	79.0	**90.2**	93.4	**98.7**	92.7	**98.7**	92.6	**98.5**	92.6	**98.5**	92.5	**98.2**	93.2	**98.7**	93.2	**98.7**
RNA/Human-wt/ITA/PA83/2007/2007/G2P[4]	I2	80.3	**92.4**	79.8	**91.2**	80.0	**92.7**	80.5	**92.7**	80.2	**91.4**	96.8	**99.5**	97.5	**99.5**	97.4	**99.2**	97.4	**99.2**	97.3	**99.0**	97.4	**99.5**	97.4	**99.5**
RVA/Human-wt/BEL/B1711/2002/G6P[6]	I2	79.5	**92.2**	79.1	**90.9**	79.4	**92.4**	79.9	**92.4**	79.8	**91.2**	97.0	**99.7**	99.1	**99.7**	99.0	**99.5**	99.0	**99.5**	98.9	**99.2**	98.2	**99.7**	98.2	**99.7**
RVA/Human-wt/IND/TK119/2003/GxP[x]	I2	79.8	**92.2**	79.3	**90.9**	79.7	**92.4**	80.2	**92.4**	80.1	**91.2**	97.2	**99.7**	98.3	**99.7**	98.2	**99.5**	98.2	**99.5**	98.2	**99.2**	98.7	**99.7**	98.7	**99.7**
RVA/Human-wt/ZMB/MRC-DPRU3463/2009/G8P[4]	I2	78.9	**91.9**	78.5	**90.7**	79.0	**92.2**	79.2	**92.2**	79.4	**90.9**	98.3	**99.5**	96.4	**99.5**	96.3	**99.2**	96.3	**99.2**	96.2	**99.0**	96.1	**99.5**	96.1	**99.5**
RVA/Human-wt/THA/CMH008/05/2005/G2P[x]	I2	79.7	**91.9**	79.3	**90.7**	79.6	**92.2**	80.1	**92.2**	79.5	**90.9**	97.6	**99.5**	97.3	**99.5**	97.2	**99.2**	97.2	**99.2**	97.1	**99.0**	97.1	**99.5**	97.1	**99.5**
RVA/Human-tc/USA/US1205/1997/G9P[6]	I2	79.3	**91.4**	78.9	**90.2**	79.2	**91.4**	79.6	**91.2**	79.3	**89.9**	97.3	**98.2**	96.7	**98.2**	96.6	**98.0**	96.6	**98.0**	96.6	**97.7**	96.7	**98.2**	96.7	**98.2**
RVA/Human-wt/BGD/RV176/2000/G12P[6]	I2	79.8	**92.2**	79.3	**90.9**	79.7	**92.4**	80.2	**92.4**	80.1	**91.2**	97.1	**99.7**	98.2	**99.7**	98.2	**99.5**	98.2	**99.5**	98.1	**99.2**	98.7	**99.7**	98.7	**99.7**
RVA/Human-tc/JPN/AU-1/1982/G3P[9]	I3	79.2	**92.2**	78.8	**90.9**	79.1	**92.4**	78.8	**91.9**	78.0	**89.7**	81.7	**94.5**	81.7	**94.5**	81.8	**94.7**	81.8	**94.7**	81.7	**94.5**	81.5	**94.5**	81.5	**94.5**
RVA/Avian-tc/JPN/PO-13/1983/G18P[17]	I4	71.6	**74.3**	71.2	**73.3**	71.7	**74.1**	71.7	**73.6**	71.2	**74.6**	71.3	**74.3**	71.6	**74.3**	71.6	**74.3**	71.6	**74.3**	71.5	**74.1**	71.8	**74.3**	71.8	**74.3**
RVA/Horse-tc/USA/FI-14/1981/G3P[12]	I6	83.5	**94.0**	83.1	**92.7**	83.9	**94.7**	83.9	**94.0**	79.9	**90.4**	80.2	**92.4**	80.6	**92.4**	80.6	**92.7**	80.6	**92.7**	80.5	**92.4**	80.9	**92.4**	80.9	**92.4**
RVA/Horse-tc/GBR/L338/1991/G13P[18]	I6	83.7	**95.0**	83.3	**93.7**	83.8	**95.7**	83.7	**95.5**	80.1	**91.2**	79.4	**92.4**	80.3	**92.4**	80.3	**92.7**	80.3	**92.7**	80.2	**92.4**	80.0	**92.4**	80.0	**92.4**
RVA/Murine-tc/USA/ETD_822/XXXX/G16P[16]	I7	77.0	**91.2**	76.6	**89.9**	77.7	**91.4**	77.2	**91.4**	75.7	**89.4**	79.4	**95.2**	78.9	**95.2**	79.0	**95.5**	79.0	**95.5**	78.9	**95.2**	79.2	**95.2**	79.2	**95.2**
RVA/Human-tc/KEN/B10/1987/G3P[2]	I9	79.8	**91.2**	79.3	**89.9**	79.9	**91.4**	79.6	**91.4**	78.8	**89.7**	81.9	**95.2**	81.9	**95.2**	82.0	**95.0**	82.0	**95.0**	81.9	**94.7**	82.4	**95.2**	82.4	**95.2**
RVA/Avian-tc/DEU/02V0002G3/2002/G19P[30]	I11	70.6	**70.8**	70.2	**69.8**	70.4	**70.5**	70.5	**70.8**	71.8	**72.0**	69.6	**71.8**	69.4	**71.8**	69.4	**71.8**	69.4	**71.8**	69.3	**71.5**	69.8	**71.8**	69.8	**71.8**
RVA/Human-wt/ECU/Ecu534/2006/G20P[28]	I13	75.1	**83.9**	74.7	**83.1**	75.8	**84.1**	75.3	**83.9**	76.0	**83.6**	75.7	**83.1**	75.4	**83.1**	75.6	**83.1**	75.6	**83.1**	75.5	**82.9**	75.7	**83.1**	75.7	**83.1**
RVA/Pig-wt/CAN/CE-M-06-0003/2006/G2P[27]	I14	80.0	**89.9**	79.6	**88.7**	80.3	**90.7**	80.1	**90.2**	80.6	**89.4**	79.6	**88.9**	79.3	**88.9**	79.3	**89.2**	79.3	**89.2**	79.3	**88.9**	79.3	**88.9**	79.3	**88.9**
RVA/Bat-wt/KEN/KE4852/07/2007/G25P[6]	I15	78.0	**90.9**	77.6	**89.7**	78.5	**91.2**	78.4	**91.2**	77.7	**89.7**	80.7	**93.2**	80.9	**93.2**	80.9	**93.2**	80.9	**93.2**	80.8	**92.9**	81.2	**93.2**	81.2	**93.2**
RVA/Simian-tc/USA/TUCH/2002/G3P[24]	I16	79.6	**91.2**	79.2	**89.9**	79.8	**91.4**	80.2	**91.4**	80.3	**89.7**	81.4	**95.2**	81.6	**95.2**	81.7	**95.0**	81.7	**95.0**	81.8	**94.7**	81.8	**95.2**	81.8	**95.2**
