# Supplementary material for: Genetic diversity of rotavirus genome segment 6 (encoding VP6) in Pretoria, South Africa
Source: Springerplus. 2014 Apr 5;3:179. doi: 10.1186/2193-1801-3-179 (PMC4000354; doi:10.1186/2193-1801-3-179)
Supplement: Supplementary file 2 — Additional file 2: Comparison of deduced amino acid sequences of VP6 proteins of South African strains to strain RF. Gray areas indicates boundaries of group specific antigenic regions (antigenic site I (32-64), antigenic site II (155-167), antigenic site III (208-274) and antigenic site IV (380-397). Residues 228-240 (Gray box area) indicates hyper variable region, residues 172, 296-300, 305 and 310 (plain box) are Subgroup I (SGI) and residues at position 306 is a Subgroup II (SGII). (DOC 145 KB) [file 40064_2013_888_MOESM2_ESM.doc]

**Supplementary data 2:** Comparison of deduced amino acid sequences of VP6 proteins of South African strains to strain RF. Gray areas indicates boundaries of group specific antigenic regions (antigenic site I (32-64), antigenic site II (155-167), antigenic site III (208-274) and antigenic site IV (380-397). Residues 228-240 (Gray box area) indicates hyper variable region, residues 172, 296-300, 305 and 310 (plain box) are Subgroup I (SGI) and residues at position 306 is a Subgroup II (SGII).
